# Ischemic preconditioning maintains immunoreactivities of glucokinase and glucokinase regulatory protein in neurons of the gerbil hippocampal CA1 region following transient cerebral ischemia

**DOI:** 10.1186/2197-425X-3-S1-A775

**Published:** 2015-10-01

**Authors:** JH Cho, CW Park, TG Ohk, MC Shin, YS Kim, MH Won

**Affiliations:** Kangwon National University, Emergency Medicine, Chuncheonsi, Korea, Republic of; Kangwon National University, Neurobiology, Chuncheonsi, Korea, Republic of

## Introduction and objectives

Glucokinase (GK) plays a key role in the control of blood glucose homeostasis. In the present study, we investigated the effect of ischemic preconditioning (IPC) on immunoreactivities of GK and its regulatory protein (GKRP) following 5 min of transient cerebral ischemia in gerbils.

## Methods

The gerbils were randomly assigned to 4 groups (sham-operated-group, ischemia-operated-group, IPC plus (+) sham-operated-group and IPC+ischemia-operated-group). IPC was induced by subjecting the gerbils to 2 min of ischemia followed by 1 day of recovery.

## Results

In the ischemia-operated-group, a significant loss of neurons was observed in the stratum pyramidale (SP) of the hippocampal CA1 region (CA1) at 5 days post-ischemia; however, in the IPC+ischemia-operated-group, neurons in the SP were well protected. In the immunohistochemical study, immunoreactivities of GK and GKRP in neurons of the SP were distinctively decreased in the CA1, not CA2/3, from 2 days post-ischemia, and hardly detected in the SP at 5 days post-ischemia. In the IPC+ischemia-operated-group, immunoreactivities of GK and GKRP in the SP of the CA1 were similar to those in the sham-group.

## Conclusions

In brief, our findings show that IPC dramatically maintains immunoreactivities of GK and GKRP in neurons of the SP of the CA1 after ischemia-reperfusion and indicate that GK and GKRP may be necessary for neurons to survive against transient cerebral ischemia.

